# Analysis of Anti-Jamming Performance of HF Access Network Based on Asymmetric Frequency Hopping

**DOI:** 10.3390/s25092950

**Published:** 2025-05-07

**Authors:** Ruijie Duan, Liang Jin, Xiaofei Lan

**Affiliations:** National Digital Switching System Engineering and the Technological Research and Development Center, Information Engineering University, Zhengzhou 450001, China; jerryduan0206@outlook.com (R.D.); xiaofeilan1618@outlook.com (X.L.)

**Keywords:** communication anti-jamming, HF access network, asymmetric frequency hopping, fixed-frequency communication, two-dimensional Markov queuing model, dynamic spectrum management

## Abstract

The primary focus of this paper lies in addressing the inadequate anti-dynamic jamming capability of the link layer within high-frequency (HF) access networks. To this end, we propose the incorporation of asymmetric frequency-hopping (AFH) technology within the wireless communication segment of HF access networks. This innovation aims to supersede the existing fixed-frequency and frequency-hopping communication methodologies, ultimately enhancing the network’s resilience against dynamic jamming. Moreover, we undertake a modeling analysis to delve into the ramifications of asymmetric frequency-hopping communication in dynamic jamming environments. This modeling framework serves to elucidate the dynamics of user spectrum occupation and jamming occurrences. Our proposed methodology leverages a two-dimensional Markov queuing model, equipped with a single server, for the purpose of managing the spectrum allocation within HF access network subnets. Consequently, the base station gains the capability to dynamically manage and adjust the available spectrum in real time, thereby effectively mitigating mutual jamming among users and facilitating the seamless implementation of asymmetric frequency hopping in HF access networks. Lastly, we conduct a simulation analysis to evaluate the changes in anti-jamming performance indices within the HF access network. This analysis compares the merits and demerits of utilizing fixed-frequency, frequency-hopping, and asymmetric frequency-hopping communication techniques. Our findings conclusively demonstrate that the integration of asymmetric frequency-hopping technology can significantly reduce outage and mutual jamming rates within HF access network subnets, thereby substantially bolstering their anti-jamming prowess.

## 1. Introduction

The high-frequency (HF) access network [1,2] is a network facilitating access functions through the utilization of HF wireless propagation. Users are connected to access base stations via HF links, which are interconnected through an existing basic wired network. This network offers several advantages, including the seamless integration of mobile and fixed users, adaptive access capabilities, and high accessibility. In this study, high frequency refers to the 3–30 MHz radio band defined by the ITU, widely used for long-distance wireless communication via ionospheric propagation, which faces unique challenges such as multi-path fading and jamming vulnerability [3]. However, it is not devoid of limitations, including geographical user location, propagation conditions, human jamming, equipment with the same address, shielding conditions, and other significantly varying factors [4]. Notably, in most cases, the electromagnetic environment and available frequencies of communication parties are asymmetric, and the link layer exhibits limited adaptability to time-varying environments [5,6]. This is particularly evident in harsh electromagnetic environments where jamming is constantly changing. Hence, the resilience of the HF access network’s link layer to dynamic jamming becomes imperative.

Consequently, the existing literature [2,7] has proposed the implementation of technology within HF access networks to enhance their link layer’s anti-jamming capacity. Asymmetric frequency hopping (AFH) can be characterized as a multi-task intelligent communication method that integrates frequency-hopping service transmission, jamming perception, transmission quality assessment, and dynamic spectrum access. This is accomplished by leveraging the HF radio’s own equipment and network resources during frequency-hopping service transmission [8]. This approach encompasses the comprehensive adoption of technologies, such as online autonomous sensing of the electromagnetic environment, analysis and evaluation of frequency-hopping frequency transmission quality, and autonomous asymmetric configuration and dynamic adjustment of authorized spectrum [9].

AFH demonstrates robust anti-jamming properties [10,11,12]. When each of the two communication transmitters has access to a single frequency point, asymmetric spectrum configuration frequency-hopping technology facilitates single-frequency point communication. Consequently, users of the HF access network leveraging AFH technology experience uninterrupted communication when accessing the base station. Furthermore, the HF access network exhibits extensive coverage, potentially spanning globally. The subnets are geographically dispersed, and the occurrence of jamming is largely independent of the subnet’s communication process, adhering to Markovian characteristics.

This paper proposes treating users and jamming in the subnet as two distinct customer classes. The management of the base station and spectrum utilization are analogized to servers accommodating users and jamming, with jamming having higher priority. If a frequency band unused for user communication is jammed, new users cannot access the base station. Similarly, if a band in use is jammed, the user loses the ability to communicate on that band. The proposed model encapsulates the dynamic interplay between users and jamming spectrum occupation within a two-dimensional Markov queuing framework (M/M/1-AFH-HFAH), encompassing two distinct customer types and a single-server class. The arrivals of jamming and users are mutually independent and follow exponential distributions. The base station dynamically manages and adjusts the available spectrum in real time to prevent mutual jamming among subnet users.

The dynamic spectrum management model (M/M/1-AFH-HFAH) introduced in this paper diverges from the traditional M/M/1 model [13,14,15]. Herein, the process of jamming reaching the affected spectrum is considered the arrival of higher-priority customers, and the base station serves these customers exclusively. During service, if a new user arrives, communication access is denied. Conversely, if a new jamming signal arrives, the ongoing service is promptly terminated, and the spectrum is occupied by the new jamming signal. This study also examines the impact of jamming on user access to the base transceiver station (BTS) and queuing. Modeling is employed to elucidate the processes of jamming and spectrum occupation by users. Consequently, the base station can manage and adjust the available spectrum in real time, effectively mitigating mutual jamming among users and supporting the application of AFH in HF access networks. The model elucidates methodologies for calculating the user communication interruption rate, mutual jamming rate, and network throughput. The anti-jamming performance indices of the HF access network are analyzed through simulations. The findings indicate that, when integrated with AFH technology, the proposed method reduces the outage rate and mutual jamming rate in the HF access network subnet, thereby enhancing its anti-jamming capability.

The core contributions of this paper are as follows: (1) the integration of AFH to enhance the anti-dynamic jamming capability of HF access networks; (2) the summarization and proposal of the spectrum management method and requirements of the HF access network based on AFH; (3) the development of a two-dimensional Markov queuing model for real-time spectrum allocation, reducing user mutual jamming and outage rates; (4) the simulation-based validation showing that the proposed method outperforms traditional fixed-frequency and frequency-hopping approaches in harsh electromagnetic environments.

This paper proceeds as follows: Section 1 introduces the challenges of HF access networks and presents the integration of AFH as the proposed solution. Section 2 introduces the background of AFH and HF access network architecture, organizing the relevant literature on spectrum management and queuing theory. Section 3 summarizes and proposes the spectrum management method and the spectrum management requirements of the HF access network based on AFH. Section 4 formulates the dynamic spectrum management problem, proposing the M/M/1-AFH-HFAN model to model user–jamming interactions. Section 5 presents the simulation results for the key metrics (outage rate, mutual jamming rate, and throughput), validating the proposed method. Finally, Section 6 concludes this study and outlines future research directions.

## 2. AFH, HF Access Network Fundamentals and Literature Review on Queuing Theory Models in Spectrum Management

### 2.1. AFH Technical Principles

AFH represents an intelligent anti-jamming technology rooted in a cognitive loop, characterized by its core attributes: cognition of the transmission environment and dynamic spectrum configuration. This technology plays a pivotal role in enabling HF AFH communication [1,2]. The HF communication systems often exhibit significant asymmetry in their channel time-varying characteristics, fading properties, jamming conditions, and service demands. To tackle these challenges, the transceiver frequency meter undergoes dynamic and real-time adjustments, facilitating the implementation of an asymmetric configuration for the frequency-hopping meter. Consequently, this approach enhances both the spectrum efficiency and the electromagnetic environment adaptability of the HF communication system.

The core technology of AFH is the AFH frequency meter, which means that in the HF frequency-hopping communication, both communication parties detect the local receiving channel, respectively, and the local receiving frequency meter is composed of the local non-jamming or low-jamming frequency, and the opposite end is also the same. The desired result is that the receiving frequency table of the opposite end is the same as the transmitting frequency table of the local end; the local receiving frequency meter is the same as the opposite transmitting frequency meter; each communication party has a different receiving and transmitting frequency table; and although the transmitter receiver frequency tables at the same end are different, they are all subsets of the original frequency-hopping frequency tables. This result truly reflects the natural situation of an HF channel, that is, HF communication can adapt to an HF channel to the maximum extent and make use of all the available HF channel resources [16].

**Figure 1:**
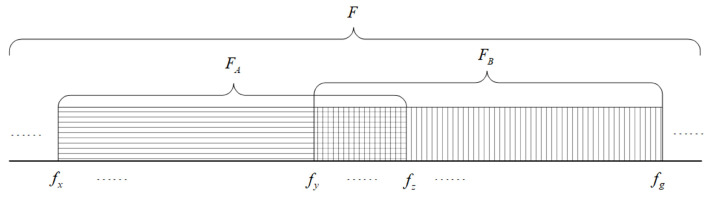
Schematic diagram of frequency selection of asymmetric frequency meter.

Assume that the same original frequency meter *F* used by both communication parties *A* and *B* is composed of $\left\{f_{i}\right\}\left(i=1,2,3,\ldots \right)$. Before communication at both ends of the link, *A* and *B*, respectively, detect and analyze the receiving environment at their own ends to obtain their own good frequencies and form the receiving frequency meters $F_{A}$ and $F_{B}$, respectively, $F_{A}\in F,F_{B}\in F$. Among them, $F_{A}$ contains the frequency $f_{x}-f_{z}$, $F_{B}$ contains the frequency $f_{y}-f_{g}$, and the common good frequency of *A* and *B* is $f_{y}-f_{z}$. When using the symmetrical frequency meter, *A* and *B* can only take the frequency within the range of $f_{y}-f_{z}$ as the common frequency meter. After using an asymmetric frequency meter, $F_{A}$ is $f_{x}-f_{z}$ and $F_{B}$ is $f_{y}-f_{g}$. After an information interaction between both parties, radio stations *A* and *B* both receive with the local receiving frequency meter and send with the opposite receiving frequency meter [1], as shown in Figure 1.

**Figure 2:**
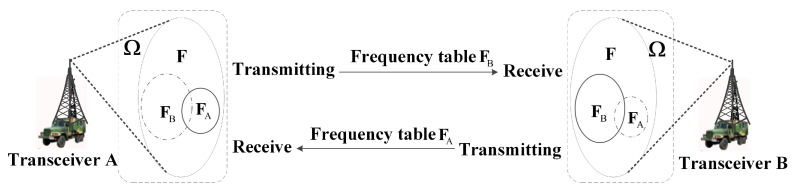
Schematic diagram of AFH communication.

For example, when users *A* and *B* communicate, *A* and *B*, respectively, detect local good frequencies and form their respective receiving frequency tables $F_{A}$ and $F_{B}$, which are all in the frequency-hopping frequency set *F*, as shown in Figure 2. In the communication process, *A* sends with frequency meter $F_{B}$ and receives with frequency meter $F_{A}$; *B* sends with frequency meter $F_{A}$ and receives with frequency meter $F_{B}$.

The symmetric frequency table is a subset of the asymmetric frequency table. The asymmetric frequency meter includes the symmetric frequency meter. After the use of asymmetric frequency meters, not only is the number of available frequencies increased but also the frequency meters in both directions are not completely the same, which can improve the spectrum utilization, frequency-hopping processing gain, anti-jamming ability, and anti-reconnaissance ability.

By effectively perceiving the electromagnetic environment in real time, AFH has demonstrated its capability to bolster the electromagnetic environment adaptability and dynamic jamming resistance of wireless communication systems through intelligent decision-making and adaptive adjustments of anti-jamming strategies. In contrast to existing frequency-hopping communication equipment, it offers an improved capacity for HF users to adapt to harsh electromagnetic environments and channel characteristics. Additionally, it augments the comprehensive defensive capabilities against reconnaissance, jamming, and access attacks.

### 2.2. Introduction of HF Access Network Based on AFH

**Figure 3:**
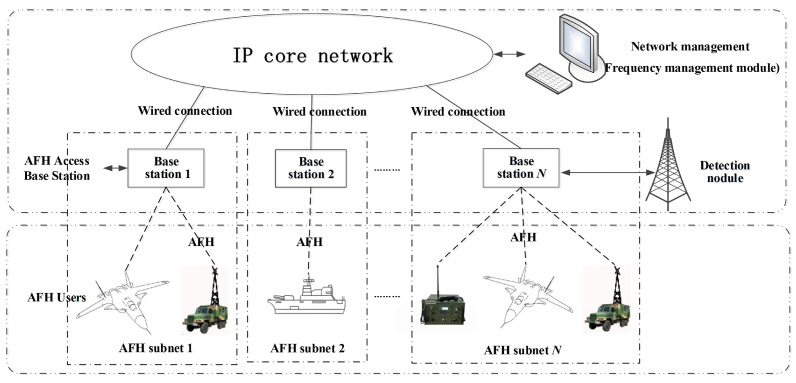
Topology diagram of HF access network using AFH.

The deployment of AFH technology within HF access networks represents a novel and groundbreaking application, specifically tailored to address the intricate and severe jamming conditions prevalent in such environments. The effectiveness of this application hinges on its capacity to augment the anti-dynamic jamming resilience and adaptability of HF access networks in hostile electromagnetic environments. The network topology is depicted in Figure 3.

To ensure that the HF access network achieves fundamental dynamic spectrum communication capabilities through AFH technology, it is crucial to maintain capabilities, such as service support, user management, security management, and regional coverage. To this end, the HF access network must undergo the following improvements:

(1) The pre-authorization strategy for frequency-hopping spectrum resources must be enhanced, and the spectrum management and service system must be refined through the application of technologies, including frequency table planning and management, the real-time monitoring of the jamming and spectrum quality, and the comprehensive assessment of frequency availability.

(2) AFH technology must be leveraged to establish a low-quality frequency shielding link between the base station and users, facilitate real-time access to available frequencies, coordinate frequency usage among various frequency meters, and implement dynamic spectrum access systems.

(3) The base station and its users form a subnet, with the base station functioning as the network and control center. The role of the control center is multifaceted: firstly, to receive spectrum allocations from the network management center; secondly, to manage spectrum resources within the subnet; and thirdly, to provide timely feedback to the network management center based on electromagnetic spectrum perception and usage within the subnet, thereby achieving closed-loop management.

Applying AFH to the HF access network will bring great performance gains. Users in each subnet (a base station in the HF access network and the mobile users it serves form a frequency-hopping subnet) use asymmetric spectrum configuration frequency-hopping technology to communicate with the base station. When there is more than one user in the subnet, multiple users compete to use the same spectrum to communicate with a base station. There are problems, such as chaotic frequency use, mutual jamming between users, and the inability to effectively support AFH communication. The problem of user jamming in the subnet is caused by the introduction of AFH technology, which is a side effect of solving the weak adaptability of the link layer of the HF access network to the time-varying environment. The current situation of the HF access network relies on the fixed spectrum allocation and access method adopted by the network management center. It is difficult to meet the spectrum access requirements of real-time changing base stations and mobile users and cannot effectively support AFH. Therefore, the spectrum management method of the HF access network subnet using AFH communication needs to be studied and solved urgently.

### 2.3. Literature Review on Queuing Theory Models in Spectrum Management

In recent years, the utilization of queuing theory models [17] has gained increasing prominence in spectrum resource management and allocation. The model has been employed to propose a high-throughput channel allocation protocol, addressing inefficient channel allocation for secondary users in complex and dynamic cognitive radio systems [17]. This protocol utilizes queuing theory to model packet transmission processes and derives performance indicators for secondary users through a Markov steady-state solution, aiming to maximize total throughput and enhance spectrum utilization. Additionally, ref. [18] proposes a spectrum allocation algorithm based on the Markov queuing theory, effectively improving throughput. Ref. [19] introduces a method for modeling the system using multi-dimensional Markov chains and deriving relevant performance indicators, yielding beneficial results. Ref. [20] presents a spectrum allocation queuing model based on queuing theory, fully considering the impact of identification errors and effectively reducing delay and improving network throughput. Ref. [21] introduces a centralized dynamic spectrum allocation scheme based on the queuing theory model, employing a straightforward and efficient spectrum allocation method, particularly suitable for wireless networks with constrained node energy, computing power, and dynamic topologies. These queuing theory-based spectrum allocation models and methods have demonstrated effective spectrum management and allocation in cognitive radio networks, offering advantages such as efficiency and ease of implementation.

These studies provide a foundation for modeling user–jamming interactions in HF subnets, but none explicitly address AFH or jamming priority. Our work extends this by integrating AFH with a two-dimensional Markov chain, accounting for both user arrivals and jamming events as competing queuing entities.

## 3. Spectrum Management Method and Requirements of HF Access Network Based on AFH

### 3.1. Decision of Self-Use Frequency at the End of HF Access Network Based on AFH

All detection information is consolidated and transmitted to the network management center for processing, where frequencies are uniformly determined across the network. This frequency management approach presents both advantages and disadvantages. The primary advantage lies in its ability to facilitate the uniform planning of the network’s frequency resources, thereby preventing potential conflicts. However, the primary drawbacks include the potential risk of non-convergence. The frequency utilization algorithm is characterized by a high level of complexity, a large number of discrete base stations, and the susceptibility of local electromagnetic environment fluctuations to impact convergence speed. These factors can hinder the efficiency of the frequency utilization algorithm, leading to the challenge of difficult convergence, which refers to the difficulty in devising an effective frequency configuration scheme.

To address these challenges, we employ a range of methodologies, including hierarchical assurance (priority), degraded assurance (quality reduction), and user reduction, to conduct iterative operations. However, this approach further compromises the timeliness of the process [2].

Moreover, users’ rapid adaptability to dynamic electromagnetic environments is limited [2]. The local electromagnetic environment for different users accessing a base station often varies, particularly as jamming is targeted at individual links, which are local (relative to the entire network) and time-varying. However, the access base station of the HF access network lacks the authority to independently configure and dynamically adjust the spectrum. Instead, it must apply to the network management center to detect and regain available frequency resources [2] (a method of addressing local issues through global actions). This process involves multiple links (reducing reliability), long time consumption (weakening the ability to adapt to dynamic environments and jamming), and low efficiency (consuming more resources, such as network bandwidth, equipment, and time). A more reasonable approach is to implement layered frequency management while adhering to centralized management, ensuring that underlying issues are resolved at the bottom level.

The utilization of passive detection technology, incorporating AFH technology, combined with comprehensive decisions on frequency availability and asymmetric spectrum configuration technology, enables the realization of terminal-independent frequency usage decisions [2]. Based on the asymmetry of the electromagnetic environment and business requirements, users can adjust the transmit–receive spectrum table in real time, facilitating asymmetric configuration of the frequency-hopping frequency table. This, in turn, enhances the spectrum efficiency and electromagnetic environment adaptability of the HF communication system.

### 3.2. Spectrum Management Method of HF Access Network Based on AFH

To effectively manage the spectrum of AFH within the HF access network, it is imperative to integrate this with the spectrum management technology inherent to the HF access network. Subsequently, the spectrum management is facilitated through the HF spectrum management system of the network management center [2]. This system is tasked with gathering business information, making frequency allocation decisions based on long-term forecasts and transmission requirements, and promptly disseminating these decisions to each base station. The approach employed in spectrum management is highly centralized.

The implementation of autonomous spectrum management stands as a crucial factor in enabling AFH. To attain autonomous spectrum management, the following principles must be adhered to: Firstly, within the authorized frequency range of the network management center, each base station and user is permitted to independently select optimal frequency points for access. Secondly, autonomous spectrum management must ensure no mutual jamming among access base stations. Thirdly, it must similarly prevent mutual jamming among users. Adherence to these principles ensures that autonomous spectrum management is both controllable and compatible with the existing HF access network system.

A management mode that promotes coexistence, mutual cooperation, sharing, and the integration of autonomous and centralized spectrum management is proposed, grounded in the distinct spectrum management paradigms of AFH and the HF access network. This approach necessitates the deployment of two distinct spectrum detection mechanisms within the network: the existing independent monitoring network and the unique sensing unit of AFH, which encompasses online spectrum monitoring and transmission channel quality analysis. To optimize the utilization of detection results from both mechanisms, repetition and conflict should be mitigated, while frequency information should be fused, complemented, and shared.

Frequency information fusion necessitates the collaboration of the following network functions:

(1) The independent monitoring network and base stations transmit monitored frequency information to the network management center via wired connections.

(2) The network management center summarizes the frequency information obtained from both the independent monitoring network and the AFH sensing unit. Based on this information, it analyzes the scope of jamming signals, the current frequency occupancy, and the fading degree of the skywave channel across various directions and frequencies. These analyses guide the periodic or as-needed updates of the frequency set allocated to each base station.

(3) The AFH sensing unit can dynamically adjust its sensing range in accordance with the new frequency set.

(4) The service and paging channels of each base station can be synchronously switched to avoid collisions.

**Figure 4:**
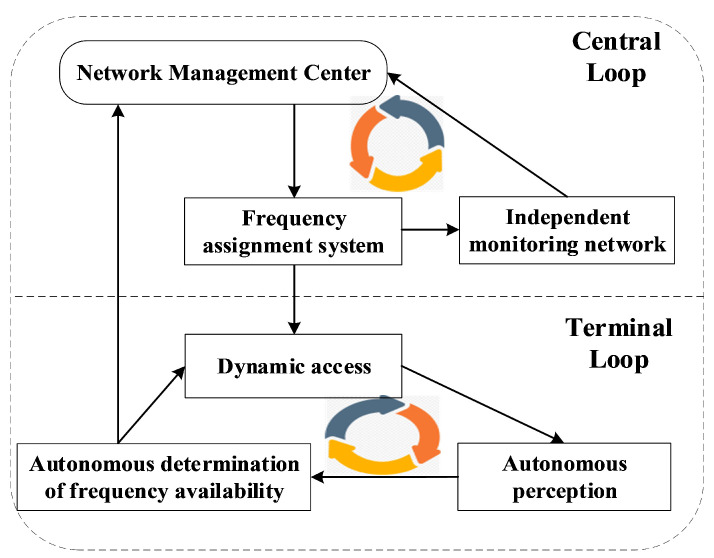
Cognitive loop composition based on autonomous perception.

It is evident that each subnet ultimately converges to a subfrequency set and undergoes dynamic changes, with corresponding adjustments to its status. Consequently, a closed-loop process of frequency information fusion emerges. The frequency cognition loop of the HF access network, augmented by the terminal autonomous sensing loop of AFH, is depicted in Figure 4.

The loop depicted above represents the decision-making allocation loop for detection in an HF access network. At this hierarchical level, frequency resources and frequency quality information undergo centralized processing by the network management center [1], a process referred to as “central loop feedback”. Conversely, the lower loop constitutes the terminal’s autonomous sensing decision-making loop, termed “terminal loop feedback”. The dynamic access of the terminal loop is constrained by the allocation results from the central loop, ensuring that frequency resource utilization among base stations does not result in mutual jamming. As illustrated in Figure 4, the terminal loop submits local decision outcomes to the network management center, serving as the reference input for frequency quality information. This approach enhances the accuracy and real-time nature of the frequency quality information source, facilitating more precise decision-making by the frequency distribution system.

To overcome the inherent challenges of HF access networks in adapting to frequency usage environments and bolster their capacity for swift adaptation to electromagnetic conditions, it is crucial to implement a closed-loop process for frequency information fusion between the top-tier network management center and the terminal asymmetric hopping subnet. The benefits of this approach are numerous. To address the limitations of the HF access network in terms of environmental adaptability for frequency usage and enhance its rapid adaptation to electromagnetic conditions, it is imperative to establish a closed-loop frequency information fusion process linking the top network management center and the terminal AFH subnet. The advantages of this methodology can be summarized as follows:

The coexistence of “central loop feedback” and “terminal loop feedback” in decision-making cognition leverages the sensing capabilities of base stations and users within the network. Evidently, a cognitive loop encompassing perception, learning, and decision-making has been established among the network management center, detection equipment, base stations, and users. The frequency allocation system can continually adjust the frequency scheme based on historical data, ensuring optimal and rational outcomes. However, the current network-wide cognitive “central loop feedback” lacks sufficient timeliness and flexibility. By integrating the dynamic configuration characteristics of AFH, the frequency allocation of “central loop feedback” and the comprehensive utilization of base station and user perception results directly realize a cognitive closed-loop system. This results in a substantial reduction in transmission links, rendering the cognitive learning process more concise and efficient.

Furthermore, the traditional single-centralized spectrum management model is poised to be supplanted by a more holistic approach, combining top-down and bottom-up management strategies. To achieve autonomous cognition, the network management center (NMC) must allocate and manage frequency resources in a manner that prevents both self-jamming and mutual jamming, ensuring that network frequency is allocated according to demand and guarantee levels. Simultaneously, the NMC assigns HF bands as backup to each base station. Within a specified frequency resource range, base stations and users can independently configure and dynamically adjust based on perceived environmental changes. These adjustment outcomes are subsequently communicated back to the network management center, serving as the foundation for subsequent decision-making and adjustment processes by the NMC.

**Figure 5:**
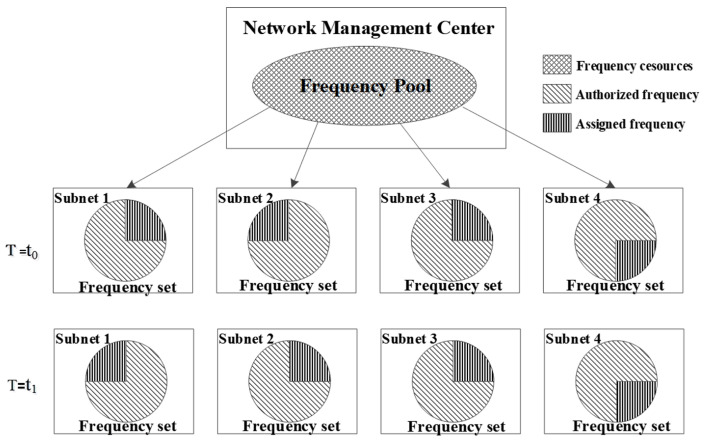
Schematic diagram of spectrum usage between subnets (FDM + TDM + SDM).

The HF access network utilizing AFH technology adopts a centralized planning and terminal-independent spectrum management approach. The utilization of frequency resources among subnets effectively constitutes a combination of frequency-division multiple access (FDMA), time-division multiple access (TDMA), and space-division multiple access (SDMA). The schematic diagram is depicted in Figure 5.

The implementation of AFH technology has exerted a profound influence on spectrum utilization, with two key aspects standing out prominently.

The network management center consistently allocates frequency sets from the frequency pool to each base station, encompassing frequencies for both the paging channel and the service channel. The service channel frequency can be designated as a fixed frequency, a series of transmit frequency tables, or an asymmetric transmit frequency table. It is crucial that the frequency assigned to each base station is unique and devoid of repetition, ensuring that the frequency set is orthogonal.

Moreover, in scenarios where users concurrently access a specific base station, it can be postulated that these users, along with the accessed base station, collectively form a small-scale local frequency-hopping star network, with the base station serving as the network’s hub. The frequency resources allocated by the base station are shared among all users within the subnet. During the process of user access to the base station, to expedite the access time, users, upon demodulating the paging information, simultaneously acquire the frequency information of the service channel and the temporal information of the channel. The availability of precise temporal information is instrumental in facilitating the swift completion of frequency-hopping access, thereby augmenting system efficiency.

Based on the topology of the HF access network and the dynamic frequency characteristics of AFH technology, the spectrum management method of the HF access network entails the following requirements.

### 3.3. Spectrum Management Requirements in the Subnet of HF Access Network Based on AFH

**Figure 6:**
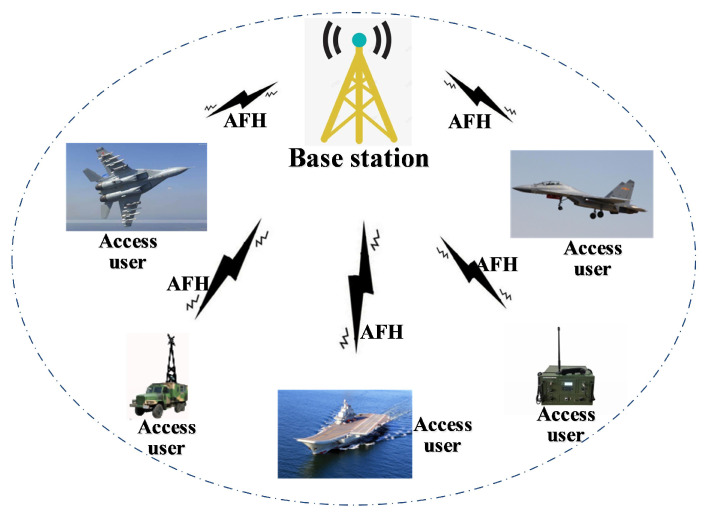
AFH star network.

When a base station and users access it simultaneously, a miniature frequency-hopping star network is established, as depicted in Figure 6.

**Figure 7:**
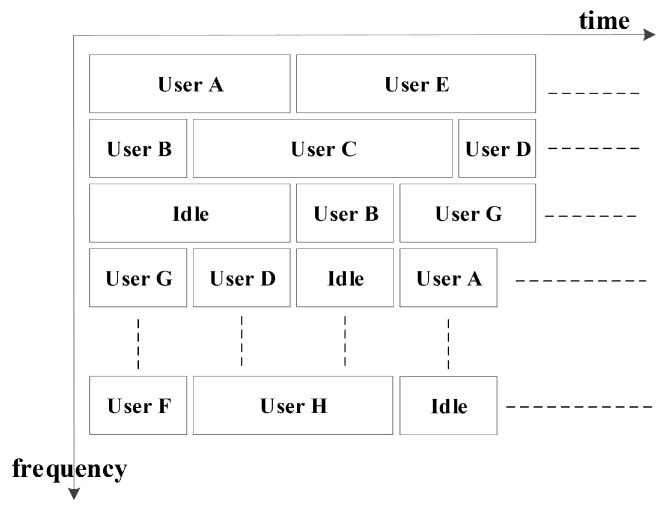
Use of frequency set in subnet (FDM + TDM).

Within the framework of the frequency-hopping star network, there exists the potential for multiple terminals at the base station to communicate with various users. The communication protocols that can be utilized encompass fixed frequency, frequency hopping, and AFH. The effective management and utilization of the network’s spectrum hinge on the implementation of frequency-division multiplexing (FDM) and time-division multiplexing (TDM) of spectrum resources, as illustrated in Figure 7.

The spectrum resources assigned to the base station by the network management center constitute the allocated resources for the subnet. Users within this subnet can share these resources through the application of time-division multiplexing (TDM) and frequency-division multiplexing (FDM). In scenarios where spectrum resources are abundant, each user employs orthogonal frequency-division multiplexing (OFDM) to utilize the spectrum. Conversely, in resource-constrained environments, the primary approach involves utilizing TDM to facilitate AFH communication between the base station and users. It is important to emphasize that the service demand within the subnet remains independent of the availability of spectrum resources. In scenarios involving confrontations between allies and adversaries, the available spectrum is highly susceptible to significant jamming from opponents at any given moment, leading to dynamic fluctuations. Consequently, multiple users compete to utilize the same spectrum for communication with the base station. This dynamic environment poses numerous challenges, including chaotic frequency utilization, mutual jamming among users, and the inability to effectively support AFH communication. Therefore, there is an urgent need to study and address the spectrum management method for AFH subnetworks in HF access networks using AFH communication.

The base station, serving as the central hub of the network, plays a crucial role in detecting, sensing, managing, and providing feedback regarding the subnet’s spectrum resources. This pivotal position can be leveraged to tackle the challenges of frequency confusion and mutual jamming among users within the subnet through the implementation of effective methodologies.

## 4. Dynamic Spectrum Management Model for HF Access Subnet Based on M/M/1/N Priority Queuing

### 4.1. Dynamic Spectrum Management Model

The subnet is modeled as an M/M/1/N queuing system, where the base station acts as the single server managing *N* shared frequency bands. Two customer types are considered: high-priority jamming events and normal-priority user sessions, with jamming preempting user sessions when targeting occupied bands.

The communication process of users in the subnet subject to jamming is modeled as a two-dimensional Markov queuing process comprising two customer types and a single server. The jamming to the subnet can be regarded as a Poisson process with an arrival rate of $\lambda_{g}$, and the time of jamming coverage spectrum is a random variable of exponential distribution with a mean value of ${\mu_{g}}^{-1}$. The communication between the user and the base station in the subnet can be regarded as a Poisson process with an arrival rate of $\lambda_{y}$, and the communication time between the user and the base station follows an exponential distribution with a mean of ${\mu_{y}}^{-1}$. It can be observed that both jamming and user arrival are independent of each other and obey exponential distribution.

The system state $\left\{E_{i,j}\right\}$ represents *i* jammed bands and *j* user-occupied bands, with 0≤i+j≤N. *N* denotes the total number of frequency bands. Jamming events arrive at rate $\lambda_{g}$ and occupy a band for ${\mu_{g}}^{-1}$ seconds; user sessions arrive at rate $\lambda_{y}$ and occupy a band for ${\mu_{y}}^{-1}$ seconds.

Jamming events have preemptive priority: when a jammer targets a user-occupied band, the user session is immediately terminated, and the state transitions from $\left\{E_{i,j}\right\}$ to $\left\{E_{i+1,j-1}\right\}$.

The two-dimensional state $\left\{E_{i,j}\right\}$ meets the requirements of $\left\{\left(i,j\right)\in E_{i,j}|\left(0\leq i,j\leq N\right)\right\}$. The balance probability corresponding to this state is $P_{i,j}$. Let $P_{i,j}$ be the statistical balance joint probability that *i* frequency bands are covered by jamming at any instant, and *j* frequency bands are being accessed by users.

The transition rate from state $\left(i,j\right)=i\cdot \mu_{g}+j\cdot \mu_{y}+\frac{N-i}{N}\lambda_{g}+\frac{N-i-j}{N}\lambda_{y}$. The transition rate from state $\left(i+1,j\right)$ to state $\left(i,j\right)=\left(i+1\right)\mu_{g}$. The transition rate from state $\left(i,j+1\right)$ to state $\left(i,j\right)=\left(j+1\right)\mu_{y}$. The transition rate from state $\left(i-1,j\right)$ to state $\left(i,j\right)=\frac{N-i-j+1}{N}\lambda_{g}$. The transition rate from state $\left(i,j-1\right)$ to state $\left(i,j\right)=\frac{N-i-j+1}{N}\lambda_{y}$.

For the state $\left(i,j\right)$, the probability flow into the state is equal to the probability flow out of it. The probability flow of the outflow from state $\left(i,j\right)$ is denoted as $\left(i\mu_{g}+j\mu_{y}+\frac{N-i}{N}\lambda_{g}+\frac{N-i-j}{N}\lambda_{y}\right)P_{i,j}$, while the probability flow of the inflow into state $\left(i,j\right)$ is $\left(i+1\right)\mu_{g}P_{i+1,j}+\left(j+1\right)\mu_{y}P_{i,j+1}+\frac{N-i-j+1}{N}\lambda_{g}P_{i-1,j}+\frac{N-i-j+1}{N}\lambda_{y}P_{i,j-1}$. Consequently, the steady-state balance equation is as follows:

(1) $$\begin{array}{cc} \; & \left(i\mu_{g}+j\mu_{y}+\frac{N-i}{N}\lambda_{g}+\frac{N-i-j}{N}\lambda_{y}\right)P_{i,j}\\ \; & =\left(i+1\right)\mu_{g}P_{i+1,j}+\left(j+1\right)\mu_{y}P_{i,j+1}+\frac{N-i-j+1}{N}\lambda_{g}P_{i-1,j}+\frac{N-i-j+1}{N}\lambda_{y}P_{i,j-1} \end{array}$$

In the event that there are *c* frequency bands present within the subnet and *k* of these are occupied, the probability $P_{k}$ can be calculated using the binomial theorem.

(2) $$\begin{array}{c} P_{k}=\sum_{i+j=k}P_{i,j}=\sum_{i+j=k}\frac{\rho_{g}^{i}}{i!}\cdot \frac{\rho_{y}^{j}}{j!}\cdot c \end{array}$$

In this context, $\rho_{g}=\frac{\lambda_{g}}{\mu_{g}}$ and $\rho_{y}=\frac{\lambda_{y}}{\mu_{y}}$ represent the load on the subnet caused by jamming and user arrival, respectively.

Then, $P_{i,j}=\frac{\rho_{g}^{i}}{i!}\cdot \frac{\rho_{y}^{j}}{j!}\cdot c$ is substituted into the balance Equation (1). The arrival rates are scaled by $\frac{N-i}{N}$ and $\frac{N-i-j}{N}$, which approximate to 1 for large *N*. The service rates $\mu_{g}$ and $\mu_{y}$ are assumed to be proportional to the number of occupied channels, a common approximation in priority queueing systems.

According to normalization conditions, we have the following:

(3) $$\begin{array}{c} c=\frac{1}{\sum_{i=0}^{N}\sum_{j=0}^{N-i}\frac{\rho_{g}^{i}}{i!}\cdot \frac{\rho_{y}^{j}}{j!}} \end{array}$$

Then, *c* is substituted into the product-form solution and we aggregate over all *i* and *j* such that i+j=k. The final expression for $P_{k}$ is as follows:

(4) $$\begin{array}{c} P_{k}=\sum_{i=0}^{k}\frac{\rho_{g}^{i}}{i!}\cdot \frac{\rho_{y}^{k-i}}{\left(k-i\right)!}\cdot c=\frac{\sum_{i+j=k}\frac{\rho_{g}^{i}}{i!}\cdot \frac{\rho_{y}^{j}}{j!}}{\sum_{i=0}^{N}\sum_{j=0}^{N-i}\frac{\rho_{g}^{i}}{i!}\cdot \frac{\rho_{y}^{j}}{j!}} \end{array}$$

In the event that the jamming is blocking jamming, the jamming degree should be set as $\delta ,\delta \left(0\leq \delta \leq 1\right)$. When δ=1, this indicates broadband blocking jamming. When 0<δ<1, it is partial band jamming. Finally, when δ=0, it is no jamming.

In the event of deterioration of the electromagnetic spectrum, with 0≤δ≤1, and the jamming covering the user’s communication frequency band, then i+j≤N and j≥1. The state $\left(i,j\right)$ in the subnet is transferred to the state $\left(i+1,j-1\right)$ with probability $\frac{j}{N-i}\lambda_{g}$; if the jamming covers the idle frequency band, the state $\left(i,j\right)$ is transferred to the state $\left(i+1,j\right)$ with probability $\frac{N-i-j}{N-i}\lambda_{g}$. In the event that i+j=N, the subnet is in a blocked state, new users are unable to gain access to communication, and the network state remains unchanged, given that the user’s communication frequency band is covered by the jamming.

As the HF access network is managed centrally, the queuing system can be implemented jointly by the network management centre and the base station as a virtual queue. In order to ensure optimal performance, the queue length should be set to a minimum of the maximum number of supported users, while the maximum queuing time should be set to the average time for user communication.

The communication process of the HF access network can be described as follows:

(1) The HF access network uses fixed-frequency communication, which refers to the maximum jamming signal ratio that the communication receiver can withstand, without regard to the jamming bandwidth. According to the characteristics of fixed frequency communication, its jamming tolerance [21] (actual anti-jamming capability) is defined as follows: For the sake of calculation convenience, the jamming tolerance of set frequency communication is approximately equal to the bit error rate when the frequency point is interfered with. When the jamming reaches ($\lambda_{g}>0$, 0<δ≤1) and covers the communication band, ongoing communication will be interrupted. If a new user attempts to communicate with the BTS, the new user will be unable to access the BTS because the frequency band has been covered by jamming.

(2) The HF access network utilizes frequency-hopping communication, with the number of hopping frequencies set at *N*. The jamming tolerance of this communication is observed to range from 30% *N* to 40% *N*, exhibiting a high sensitivity to the jamming bandwidth. In this paper, the jamming tolerance of FH communication is set at 30% *N*. When the jamming reaches ($\lambda_{g}>0$, δ≥0) and covers the communication frequency band, the communication frequency covered by the jamming is δN. Conversely, if δ<0.3, the communication is normal. Conversely, if δ≥0.3, the ongoing communication will be interrupted. In the event that δ<0.3 and no other user uses this band, a new user can access the communication if they wish to access the base station through this band. However, if δ≥0.3 or a user is using this band for communication, the new user cannot access the communication.

(3) The HF access network utilizes AFH communication, with the number of available frequencies set at *N*. The user access base station communication in the subnet employs a two-dimensional Markov queuing mechanism. In the event of jamming ($\lambda_{g}>0$, δ≥0) and communication band coverage, the asymmetric spectrum configuration frequency-hopping technology can maintain communication without interruption. In the event that a new user seeks to access the BTS, it is imperative to note that this access can only be facilitated through alternative bands. The utilization of this band is precluded. In the absence of available bands, the user’s entry into the queue is initiated, and access to communication is granted only upon the availability of a free band. Furthermore, when the jamming diminishes or the communication between the user and the base station is terminated, the spectrum is released, and the user in the queue utilizes this band to access the communication. The waiting services in the queue are queued according to the principle of first come, first served (FCFS). Furthermore, if the waiting time of users in the queue surpasses the maximum permissible queueing time, they will disassociate from the subnet.

The M/M/1-AFH-HFAN model introduces polynomial complexity due to the two-dimensional state transitions. Traditional fixed-frequency systems have O(1) complexity (no dynamic allocation), while frequency hopping exhibits O(N) complexity with the hop count. Our model’s complexity is $O(N^{2})$ due to the joint user-jamming state space, manageable via matrix inversion or iterative solvers for practical *N* (e.g., N≤200). Modern computational tools (MATLAB R2024a) efficiently handle this, as demonstrated in Section 5, balancing complexity with performance gains in anti-jamming resilience.

### 4.2. Performance Index

In order to demonstrate the advantages of AFH and two-dimensional Markov queuing theory in the subnet with clarity, the following performance indicators can be used to measure.

Access delay in our model can be derived from the queuing system’s waiting time distribution. For a single-server queue with priority handling (jamming > users), the average access delay *D* for users is a function of the queue length distribution $P_{i,j}$ and the service rate $\mu_{y}$. We will add a mathematical derivation of *D* using Little’s Law and the steady-state probabilities established in Section 4.1, showing that $D=\frac{\lambda_{y}}{\mu_{y}\left(\mu_{y}-\lambda_{y}\right)}$ under light jamming conditions, increasing with higher $\lambda_{g}$ due to spectrum preemption.

Fixed-frequency communication exhibits infinite delay upon jamming (no queuing) and traditional frequency hopping shows high delay when δ>30% (due to failed hops), while our proposed method maintains bounded delay via queuing and dynamic reallocation.

The communication interruption rate is defined as the probability of communication interruption caused by jamming during the communication between the user and the base station. That is, it is the ratio of the number of users who fail to complete the service transmission in a unit time to the number of all users who attempt to achieve the service transmission. Let us say that at a certain moment, the network management center allocates *N* band to the subnet. $P_{i,j}$ means that at any instant, there is *i* band covered by jamming, *j* band has the statistical balanced joint probability used by users, and i+j=N, with the sum of its balanced joint probability $\sum_{i=N-j}^{N}P_{i,j}=1$. The proportion of the frequency band occupied is as follows: $\sum_{i=N-j}^{N}P_{i,j}$. Then, the outage rate can be expressed as follows:

(5) $$\begin{array}{c} \varphi =\frac{\sum_{i=N-j}^{N}P_{i,j}}{\sum_{i=0}^{N}\sum_{j}^{N-i}P_{i,j}} \end{array}$$

The numerator $\sum_{i=N-j}^{N}P_{i,j}$ aggregates the probabilities of all system states $\left(i,j\right)$ where jamming causes communication outages.

The denominator $\sum_{i=0}^{N}\sum_{j}^{N-i}P_{i,j}$ is the normalization term, representing the total probability of all possible system states. It sums $P_{i,j}$ over all valid $\left(i,j\right)$ pairs where *i* and *j* satisfy the capacity constraint i+j≤N. This ensures the outage rate is a normalized probability measure, bounded between 0 (no outage) and 1 (complete outage).

In the event that the degree of jamming is being impeded by another form of jamming, the resulting jamming degree is $\delta \left(0\leq \delta \leq 1\right)$. Furthermore, the communication outage rate can be calculated using the following method:

(1) It is assumed that the outage rate of the channel in the absence of jamming is φ=0.

(2) In the case of a frequency-hopping frequency table comprising *N* frequency points, when the electromagnetic spectrum is not subject to jamming (jamming degree δ=0), the bit error rate from the sender to the receiver and from the receiver to the sender is $G_{i}\left(i\leq N\right)$. In the event that the electromagnetic spectrum is subject to jamming (δ=1), the bit error rate is $H_{i}\left(i\leq N\right)$, resulting in an interruption rate as follows:

(6) $$\varphi =\delta G_{i}^{2}+\left(1-\delta \right){H_{i}}^{2}$$

where δ is the weight factor for the jamming impact (0≤δ≤1).

This is for frequency point *i*. Furthermore, the jamming tolerance of frequency-hopping communication is 30%, whereby when the jamming is less than 30%, the communication signal can be identified; conversely, when the jamming is more than 30%, the communication signal cannot be identified. The interruption rate of frequency-hopping communication is as follows:

(7) $$\left\{\begin{array}{c} \varphi =0,\delta \leq 0.3\\ \varphi =\frac{1}{0.7N}\sum_{i=0.2N}^{N}\left[\delta G_{i}^{2}+\left(1-\delta \right){H_{i}}^{2}\right],\delta >0.3 \end{array}\right.$$

(3) Fixed-frequency communication with *N* available frequency points is characterized by an outage rate that is expressed as the average of the outage rates of all the frequency points.

(8) $$\left\{\begin{array}{c} \varphi =\delta ,\delta \leq \frac{1}{N}\sum_{i=1}^{N}H_{i}\\ \varphi =1-\frac{1}{N}\sum_{i=1}^{N}G_{i},\delta >\frac{1}{N}\sum_{i=1}^{N}H_{i} \end{array}\right.$$

(4) The AFH frequency table with *N* frequency points employs an asymmetric spectrum configuration method to configure the frequency-hopping frequency table in accordance with real-time frequency fluctuations. This enables the rapid selection of the frequency point offering the optimal transmission quality for communication, thereby reducing the break rate.

(9) $$\varphi =min{G_{i}}^{2}$$

The probability of mutual jamming caused by a newly arrived user accessing a base station when the communication is normal (i.e., not interrupted) can be calculated. The focus is on the probability of mutual jamming between two users. Let the frequency set allocated by the network management center for users *x* and *y* be Ω, and $F_{x}$ and $F_{y}$ represent the available frequency sets for users *x* and *y*, respectively. $\theta_{x,y}$ indicates the probability of mutual jamming between two users, so $\theta_{x,y}$ can be expressed as follows:

(10) $$\theta_{x,y}=\frac{\left|F_{x}\cap F_{y}\right|}{N},F_{x},F_{y}\subseteq \Omega$$

The average crosstalk rate of the subnet is as follows:

(11) $$\theta =\frac{1}{N}\sum_{x=0}^{N}\sum_{y=x+1}^{N}\theta_{x,y}$$

The term “network throughput” is used to describe the maximum amount of data that can be transmitted through a network in a given time period. It is a useful metric for assessing the overall capacity of a network. It can be defined as the quantity of data transmitted between all users and the base transceiver station within a given time period.

(12) $$B=\frac{1}{N}\sum_{i,j}^{N}P_{i,j}\sum_{k}p_{k}$$

In this context, the term $p_{k}$ is used to refer to the amount of data transmitted per unit time by a user.

## 5. Simulation and Analysis

In this paper, the influence of the HF electromagnetic environment must be considered when simulating an HF access network.

The simulation environment is designed to replicate the key characteristics of HF access networks, including ionospheric propagation effects, spectrum scarcity, and asymmetric jamming environments, as shown in Table 1.

The objective of this study is to ascertain the potential advantages that the frequency method introduced herein can confer upon HF access networks. To achieve this aim, the present paper undertakes a comparative analysis and validation of the performance characteristics of AFH in HF access networks. This analysis encompasses networks utilizing fixed frequency, conventional frequency hopping, AFH, and a two-dimensional Markov queuing model. The performance metrics under scrutiny include the interruption rate, mutual jamming rate, and network throughput during user–base station communications under conditions of jamming. The simulation parameters employed in this study are detailed in Table 2.

**Figure 8:**
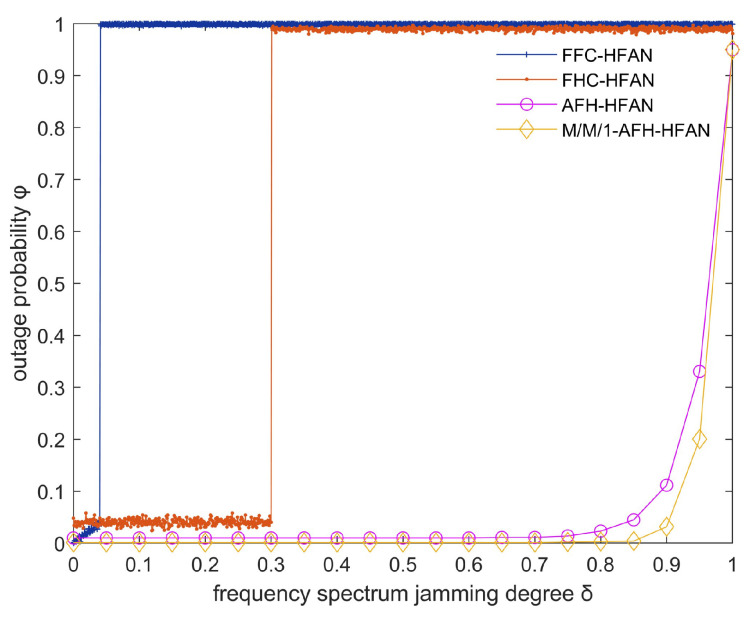
Variation in outage probability with spectrum jamming degree (δ).

**Figure 9:**
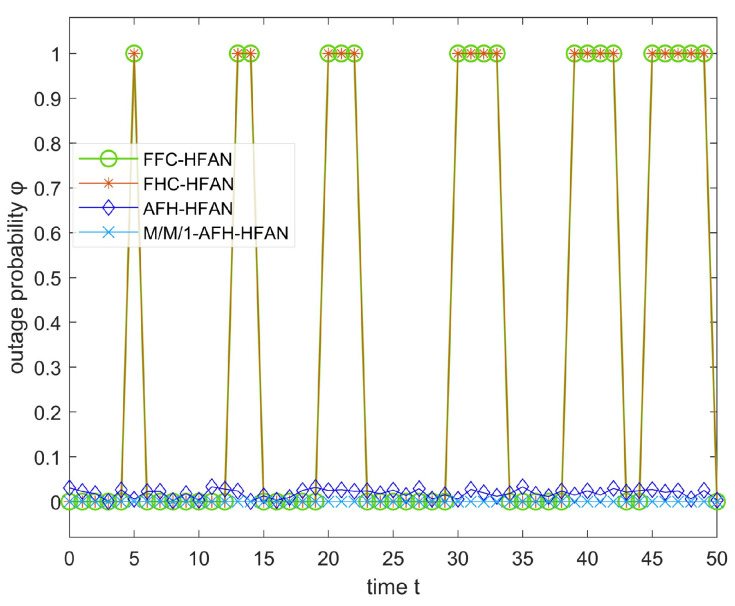
Communication interruption rate affected by jamming occupying spectrum time.

**Figure 10:**
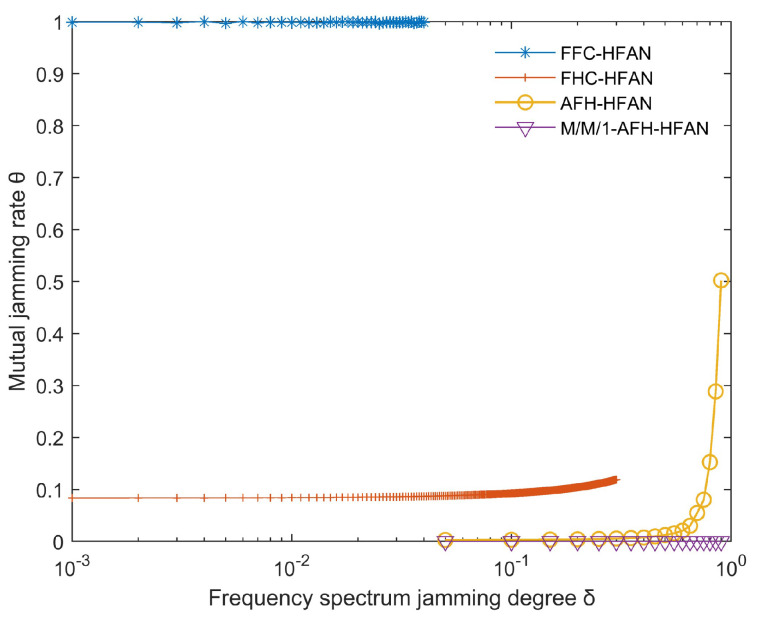
Variation in mutual jamming rate with spectrum jamming degree (δ).

**Figure 11:**
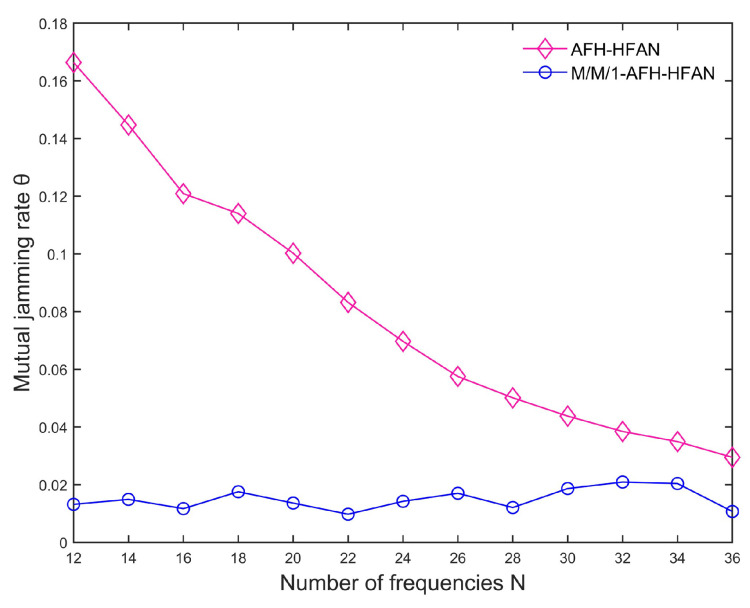
Variation in mutual jamming rate with number of frequencies.

**Figure 12:**
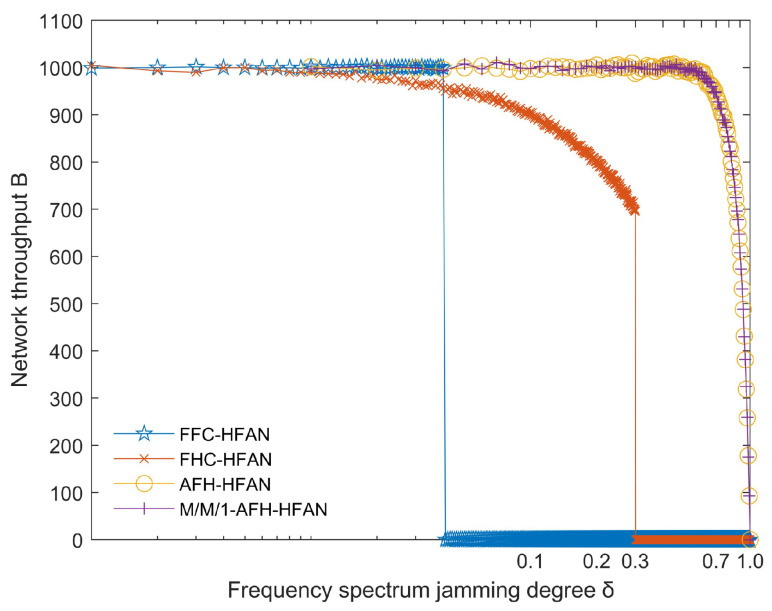
Variation in network throughput with spectrum jamming degree (δ).

The subsequent section elucidates the algorithm and its operational environment, aiming to enhance clarity in its presentation. Throughout this exposition, the terminology specific to the algorithm in question will be adopted, as outlined in Table 3. The MATLAB R2024a software is utilized to simulate and validate various performance metrics using the time scheduling method. The resultant simulation outcomes are presented in Figure 8, Figure 9, Figure 10, Figure 11 and Figure 12.

The four algorithms’ differences lie in the spectrum allocation strategy: FFC uses static frequencies, FHC uses symmetric hopping, AFH uses dynamic asymmetric tables, and M/M/1-AFH adds queuing for user contention.

Narrowband Jamming: Targets 1–3 frequencies randomly, with power 10 dB above the noise floor. Wideband Jamming: Covers a contiguous frequency range (δN, where δ is the jamming degree), mimicking adaptive adversarial jamming.

FFC-HFAN: Uses a single fixed frequency (10 MHz), vulnerable to any jamming on that frequency.

FHC-HFAN: Has a hopping sequence of 10 frequencies, with a dwell time of 50 ms, tolerant to up to 30% band jamming [1].

M/M/1-AFH-HFAN: Updates frequency tables every 20 ms based on real-time sensing, with a queuing buffer size of 8 users (FCFS discipline).

### 5.1. Outage Probability Simulation and Analysis

In scenarios where a band comprises $N\left(N>>1\right)$ frequency points and the electromagnetic spectrum undergoes blocking jamming, the average outage rate of users within the subnet is simulated, as depicted in Figure 8.

As depicted in Figure 8, the following observations can be made: (1) The communication interruption rate for users employing fixed-frequency and conventional frequency-hopping communication exhibits a gradual increase with the intensification of jamming (δ). Conversely, the interruption rate for users utilizing AFH communication remains relatively stable until the spectrum becomes unavailable, highlighting the superior anti-jamming capabilities of AFH compared to fixed-frequency communication.

(2) As δ increases from 0, the frequency point using fixed-frequency communication suffers jamming. At this time, user communication is interrupted, and the communication interruption rate quickly jumps to 100%.

(3) As the jamming level δ increases from 0 to 0.3, the communication interruption rate of users using frequency-hopping communication remains unchanged. This is because the interference tolerance of frequency-hopping communication is about 30%. When the δ jamming is less than 30%, the communication signal can be identified and the user communication is almost unaffected. When δ is greater than 0.3, the user communication signal using frequency-hopping communication will be indistinguishable, and the user communication interruption rate will quickly jump to 100%.

(4) The implementation of M/M/1-AFH-HFAN has the potential to significantly reduce the frequency of disruptions in user communication compared to alternative methods, including fixed frequency, conventional frequency hopping, and AFH. This underscores the superiority of M/M/1-AFH-HFAN in this particular context.

In scenarios where an adversary anticipates the communication frequency with the intent of implementing tracking jamming, the parameter δ in question assumes a notably small value at any given instant. However, this does not inherently imply a lack of effectiveness in its jamming impact. When user communication within a subnet is subjected to tracking jamming, the communication interruption rate is contingent upon both the jamming arrival rate and the duration for which the spectrum is occupied by the jamming signal, as depicted in Figure 9.

As illustrated in Figure 9, in the absence of tracking jamming, all communication modes operate normally, exhibiting a negligible outage rate. However, upon the introduction of tracking jamming, users utilizing fixed-frequency and conventional frequency-hopping communication encounter significant difficulties in maintaining communication, leading to an interruption rate of 100%. In contrast, AFH demonstrates a robust resilience against dynamic jamming, resulting in a break rate approaching zero. The primary determinant of anti-jamming capability is the communication mode, with the spectrum usage method having a negligible impact on this capacity.

In summary, AFH exhibits a notably superior anti-jamming capability compared to fixed-frequency and conventional frequency-hopping communication. Conversely, the employment of M/M/1-AFH-HFAN for HF access network subnet communication proves to be more effective than its absence, offering a straightforward and efficient solution.

### 5.2. Simulation and Analysis of User Jamming Rates in Subnets

In the scenario where a specific segment of the frequency band comprises $N\left(N>>1\right)$ multiple frequency points and a single user is engaged in communication, the simulation results for the average mutual jamming rate between that user and other users within the subnet are presented in Figure 10.

The following can be seen from Figure 10: (1) As δ increases from 0, the frequency point using fixed-frequency communication suffers jamming. At this time, the user communication is interrupted, and the mutual jamming between this user and other users will not exist. This is because fixed-frequency communication cannot change the communication frequency in real time.

(2) As δ increases from 0 to 0.3, the mutual jamming rate between the user using frequency-hopping communication and other users remains unchanged. This is because the jamming tolerance of frequency-hopping communication is about 30%. When δ is less than 30%, the communication signal can be identified and the user communication is almost unaffected. When δ is greater than 0.3, the user communication signal using frequency-hopping communication will be indistinguishable, the user communication will be interrupted, and the mutual jamming between this user and other users will not exist.

(3) The crosstalk rate of users using AFH is less than that of users using fixed frequency and frequency hopping. At the same time, using M/M 1-AFH-HFAN can significantly reduce the crosstalk rate to 0.

Figure 11 presents a comparison of the crosstalk rate among users before and after the implementation of M/M/1-AFH-HFAN. The results indicate that the crosstalk rate for users employing M/M/1-AFH-HFAN is significantly lower than that for users who do not utilize it. This is attributed to the fact that the method proposed in this paper is a first-come, first-served user access method specifically designed to mitigate mutual jamming and effectively support AFH communication without compromising the real-time detection of AFH or the dynamic configuration of the frequency table. Furthermore, when utilizing M/M/1-AFH-HFAN, the average crosstalk rate among users is observed to decrease as the number of frequency bands increases. This is because as the number of available frequencies rises, the base station allocates a broader spectrum to each user, leading to a reduction in the mutual jamming rate among users. In essence, an increase in the number of frequencies results in a decrease in the mutual jamming rate.

### 5.3. Network Throughput Simulation and Analysis of Subnets

Figure 12 illustrates that when the electromagnetic spectrum is subjected to blocking jamming, the number of users is 16 and the number of frequencies is 32.

(1) As δ increases from 0, the frequency point using fixed-frequency communication suffers jamming. At this time, the user communication is interrupted, and the network throughput quickly jumps from the highest point to 0.

(2) As δ increases from 0 to 0.3, the communication quality of users using frequency-hopping communication decreases. However, by means of error correction coding and other means, the minimum output signal-to-noise ratio required by the communication system demodulator can be achieved, maintaining basic communication, and the network throughput slowly decreases. This is because the jamming tolerance of frequency-hopping communication is about 30%. When δ is greater than 0.3, the user communication signal using frequency-hopping communication will be indistinguishable, the user communication will be interrupted, and the network throughput will quickly jump to 0.

(3) The network throughput achieved through the utilization of AFH remains high and does not begin to decline until the spectrum is severely disturbed. This demonstrates that the network throughput achieved through the utilization of AFH is markedly superior to that attained through the deployment of fixed-frequency hopping and frequency-hopping communication techniques, particularly in the context of heightened electromagnetic spectrum jamming. This is due to the fact that M/M/1-AFH-HFAN is primarily employed to address the issue of mutual jamming between users. The simulation depicted in Figure 12 solely considers the impact of the electromagnetic environment’s jamming degree on the network throughput.

## 6. Conclusions

Firstly, the inherent weakness in the link layer of the HF access network concerning its anti-dynamic jamming capability is tackled by integrating AFH technology into its wireless communication component, thereby significantly enhancing its resistance to dynamic jamming.

Secondly, the challenge posed by multiple users within a subnet competing for the same spectral segment for communication with the base station is addressed by examining the chaotic and disordered frequency behavior, inter-user jamming, and the incapacity to efficiently support AFH communication.

Thirdly, this paper introduces a dynamic spectrum management model for HF access network subnets, leveraging a single-server two-dimensional Markov queuing system. This model is analyzed to understand the unique characteristics of the dynamic spectrum management approach. In this framework, jamming and users are treated as distinct customer classes, with jamming assigned higher priority. Both classes arrive according to a Poisson process, and their spectrum occupation times follow an exponential distribution. By leveraging centralized management of the subnet spectrum, the network management center and base station can employ this method to ensure orderly frequency usage even in the presence of jamming.

Finally, a simulation analysis is conducted to compare the anti-jamming performance of AFH with fixed-frequency and conventional frequency-hopping communication, both before and after implementing the dynamic spectrum management method for HF access network subnets based on the single-server two-dimensional Markov queuing. The findings reveal that the combination of this method with AFH technology results in low communication interruption rates and mutual jamming rates within the subnet, demonstrating robust anti-jamming capabilities.

## Figures and Tables

**Table 1:** Simulation environment and HF-specific parameters.

Category	Parameter	Value	HF Network Rationale
Frequency Band	Operating frequency range	3–30 MHz (ITU-defined HF band)	Core HF communication band, subject to ionospheric reflection and multi-path fading.
Propagation Model	Ionospheric fading	Rayleigh fading with time-varying Doppler spread (0.5 Hz)	Models HF skywave propagation instability due to ionospheric turbulence.
Jamming Characteristics	Jamming type	Mixed narrowband (30% of cases) and wideband (70% of cases), with random center frequency within the HF band	Realistic HF jamming includes both targeted and blanket jamming.
Subnet Configuration	Number of users (K)	16 (dynamic arrivals, $\lambda_{y}=0.8$ users/s)	Typical small-scale subnet in HF tactical networks.
Spectrum Resources	Total available frequencies (N)	32 (3–30 MHz, 1 MHz spacing)	Limited spectrum resources in HF bands, leading to intense user competition.
Base Station Coverage	Communication range	500 km (skywave propagation model)	Typical HF single-hop communication distance.

**Table 2:** Table of simulation parameters.

Parameter	Parameter Value
Jamming degree of electromagnetic spectrum (δ)	[0,1] values in sequence
Jamming arrival rate ($\lambda_{g}$/s)	[0,1] values in sequence
User arrival rate ($\lambda_{y}$ Pieces/s)	0.8
Mean time of channel occupied by jamming (${\mu_{g}}^{-1}$/s)	4
Average user access communication (${\mu_{y}}^{-1}$/s)	3.6
Simulation time (T/s)	1000

**Table 3:** Description of simulation algorithm.

Algorithm Abbreviation	Full Name	Core Mechanism	Key Characteristics
FFC-HFAN	Fixed-Frequency Communication in HF Access Network	Uses a single static frequency for all user–base station communication. No frequency hopping or dynamic spectrum adjustment; vulnerable to narrowband jamming.	(1) Static spectrum allocation; (2) no adaptability to jamming; (3) low complexity (*O*(1)).
FHC-HFAN	Frequency-Hopping Communication in HF Access Network	Employs symmetric frequency hopping with a predefined set of *N* frequencies. Hopping sequence is fixed and identical for all users, relying on time-division multiplexing (TDM) to reduce collisions.	(1) Fixed hopping pattern; (2) moderate jamming tolerance (30% band coverage); (3) O(N) complexity.
AFH-HFAN	Asymmetric Frequency Hopping in HF Access Network	Dynamically configures independent transmit/receive frequency tables for each user using real-time spectrum sensing (Section 2.1). Adjusts hopping frequencies to avoid jammed bands but does not incorporate queuing for user contention.	(1) Asymmetric frequency tables; (2) real-time jamming avoidance; (3) no user queuing mechanism.
M/M/1-AFH-HFAN	Asymmetric Frequency Hopping with Two-Dimensional Markov Queuing Model	Combines AFH’s dynamic frequency configuration with a priority-based Markov queuing system (Section 4.1). Manages user arrivals and jamming events as competing queuing entities, queuing user requests during spectrum congestion and prioritizing jamming resolution.	(1) Asymmetric frequency tables; (2) priority handling for jamming; (3) queued user access (FCFS); (4) *O*($N^{2}$) complexity for state transitions.

## Data Availability

The original contributions presented in this study are included in this article. Further inquiries can be directed to the corresponding author.

## Abbreviations

The following abbreviations are used in this manuscript:

HF	High frequency
AFH	Asymmetric frequency hopping
FFC	Fixed-frequency communication
FHC	Frequency-hopping communication
HFAN	HF access network
BTS	Linear dichroism base transceiver station
NMC	Network management center
FCFS	First come, first served
FDM	Frequency-division multiple
TDM	Time-division multiple
SDM	Space-division multiple
